# Degradation of Benzo[a]pyrene and 2,2′,4,4′-Tebrabrominated Diphenyl Ether in Cultures Originated from an Agricultural Soil

**DOI:** 10.3390/toxics12010033

**Published:** 2024-01-01

**Authors:** Shuai Shi, Huiqian Zhang, Shuai Zhang, Lijin Yi, Gulijiazi Yeerkenbieke, Xiaoxia Lu

**Affiliations:** Ministry of Education Laboratory for Earth Surface Processes, College of Urban and Environmental Sciences, Peking University, Beijing 100871, China

**Keywords:** benzo[a]pyrene, 2,2′,4,4′-tetrabrominated diphenyl ether, degradation, culture, bacteria

## Abstract

Benzo[a]pyrene (BaP) and 2,2′,4,4′-tetrabrominated diphenyl ether (BDE-47) are common contaminants in the environment, posing a threat to the ecosystems and human health. Currently, information on the microbial metabolism of BaP and BDE-47 as well as the correlated bacteria is still limited. This research aimed to study the degradation of BaP and BDE-47 by enriched cultures originated from an agricultural soil in Tianjin (North China) and characterize the bacteria involved in the degradation. Two sets of experiments were set up with BaP and BDE-47 (2 mg/L) as the sole carbon source, respectively. The degradation of BaP and BDE-47 occurred at rate constants of 0.030 /d and 0.026 /d, respectively. For BaP, the degradation products included benzo[a]pyrene-9,10-dihydrodiol or its isomers, ben-zo(a)pyrene-7,8-dihydrodiol-9,10-epoxide, and cis-4 (8-hydroxypyrenyl-7)-2-oxo-3-butenoic acid. For BDE-47, the degradation products included 2,2′,4-tribrominated diphenyl ether (BDE-17), 2,4-dibrominated diphenyl ether (BDE-7), and hydroxylated dibromodiphenyl ether. The bacterial community structures in the original soil, the BaP culture, and the BDE-47 culture were quite different. The richness and diversity of bacteria in the two cultures were much lower than that in the original soil, and the BaP culture had higher richness and diversity than the BDE-47 culture. In the BaP culture, multiple species such as *Niabella* (23.4%), *Burkholderia-Caballeronia-Paraburkholderia* (13.7%), *Cupriavidus* (8.3%), and *Allorhizobi-um-Neorhizobium-Pararhizobium-Rhizobium* (8.0%) were dominant. In the BDE-47 culture, an unassigned species in the Rhizobiaceae was dominant (82.3%). The results from this study provide a scientific basis for the risk assessment and bioremediation of BaP and/or BDE-47 in a contaminated environment.

## 1. Introduction

Contaminations of polycyclic aromatic hydrocarbons (PAHs) and polybrominated diphenyl ethers (PBDEs) are common in a variety of ecosystems, particularly in soil and sediment [1,2]. In many farm lands near e-waste disassembly sites, PAHs and PBDEs are frequently detected as pollutants. For instance, in the farm lands near an e-waste disassembly site in East China, the total concentration of 16 priority-controlled PAHs ranged from 149.0 to 2.0 × 10^4^ µg/kg [3], and the total concentrations of 12 PBDEs ranged from 21.8 to 1300 µg/kg [4]. Among the PAHs and PBDEs, Benzo[a]pyrene (BaP) and 2,2′,4,4′-tetrabrominated diphenyl ether (BDE-47) deserve more attenuation, since they are highly toxic [5,6].

Biodegradation is an important pathway for removing BaP and BDE-47 from the soil. Many bacteria could produce enzymes like dioxygenase, hydrolase, formamidase and cytochrome P450 to degrade BaP [7]. BaP can be degraded via metabolism or co-metabolism. For instance, a bacterial strain *Staphylococcus haemoliticus* 10SBZ1A isolated from oil contaminated soils is capable of degrading BaP. This strain can survive in the presence of 4–200 μmol/L of BaP as the sole source of carbon, and it could degrade BaP at a rate of 0.8 μmol/L per day [8]. However, in most of the cases, bacteria cannot use BaP as the sole source of carbon and energy, thus they require additional growth substrates for their growth and metabolism. A study showed that the strain *Bacillus subtilis* BUM was not able to utilize BaP as the sole carbon and energy source, however, being provided with 50, 250 and 500 mg/L phenanthrene, this strain degraded BaP by 14.8%, 38.8% and 63.3%, respectively [9]. Another strategy that has been found effective for the microbial degradation of pollutants is the use of consortia. In the study of Aziz et al., *Ochrobactrum anthropi* and *Stenotrophomonas acidaminiphila* could degrade 26% and 20% of BaP after incubating for 8 days in seawater, while their consortia degraded 41% of BaP in the same period [10].

The biodegradation of BDE-47 may occur via multiple pathways. BDE-47 could be reductively debrominated to tri-BDEs (BDE-17 or 28). In a culture consisting of *Dehalococcoides* and *Desulfovibrio* spp., ortho-debromination of BDE-47 to BDE-28 took place first, and then BDE-28 was debrominated to BDE-15 (ortho-debromination) [11]. In a sediment-free enrichment culture, BDE-47 was debrominated to BDE-17 (para-debromination) and then BDE-17 was debrominated to BDE-4 (para-debromination) [12]. In anaerobic PBDE debromination microcosms, microbial populations belonging to the bacterial genera *Dehalococcoides*, *Dehalogenimonas*, and *Dehalobacter* were prevalent. Genes encoding PBDE reductive dehalogenase (RDases), i.e., pcbA1, pbrA2, pbrA3, pteA, tceA, bdeA, pcbA4, pcbA5, and mbrA were identified [13,14,15]. Metabolic degradation rather than reductive debromination of BDE-47 was also observed in sediment microcosms. BDE-47 was decreased over 30% in several of the microcosms without a significant accumulation of lower brominated products, indicating that there was likely a BDE-47 transformation process occurring other than reductive debromination [16]. BDE-47 could be used as the sole carbon source by bacteria B. cereus S1 and A. faecalis S4 [17]. Aerobic degradation of BDE-47 was different from reductive debromination with some unique key features, e.g., cleavage of aromatic ring and hydroxylation [18,19].

Currently, knowledge on the bacteria capable of metabolizing BaP and BDE-47 is still limited. This study aimed to investigate the aerobic degradation of BaP and BDE-47 by cultures originated from an agricultural soil and characterize the bacteria involved. To this end, microorganisms from the soil were cultured with either BaP or BDE-47 as the sole carbon source. The degradation products were identified, and the key bacteria were characterized. The results provide a scientific basis for the risk assessment and bioremediation of BaP and/or BDE-47 in a contaminated environment.

## 2. Materials and Methods

### 2.1. Chemicals and Soil

Solid BaP (96%), 10-deuterated pyrene (Pyr-d10), BaP-d12 (98%), BDE-47 (98%) and standard solutions of BaP, BDE-47, BDE28, BDE-17, BDE-15, BDE-7, BDE-4, BDE-3, BDE-1, PCB-141, PCB-208 (1000 µg/mL in acetone) were purchased from AccuStandard (New Haven, CT, USA). Analytical pure acetone, n-hexane, dichloromethane, yeast extract, Na_2_HPO_4_·12H_2_O, KH_2_PO_4_, MgSO_4_·7H_2_O, and NH_4_Cl were purchased from Beijing Tongguang Fine Chemical Company (Beijing, China).

The soil used in this study was originally collected from the surface of an agricultural land (117° E, 39° N) in Tianjin, North China. The soil belonged to fluvo-aquic soil, a subclass of alluvial new soil under the international soil classification system. The total organic carbon (TOC) and pH values of the original soil were 12.54 g/kg and 6.9, respectively. The concentrations of polycyclic aromatic hydrocarbons and polybrominated diphenyl ethers were below the detection limits in the original soil. The soil (200 g) was spiked with BaP and BDE-47 (about 30 mg/kg for each compound) in a 280 mL glass jar and incubated aerobically with the jar mouth covered with perforated sealing film for three months. At the end of the incubation, the concentrations of BaP and BDE-47 in the soil were 10.97 and 8.28 mg/kg, respectively. The incubated soil was used as an inoculation for the BaP and BDE-47 degrading experiments.

### 2.2. Experimental Setup

About 5 g of the above-mentioned soil was taken from the incubation jar and placed into a 50 mL centrifuge tube containing 20 mL ultrapure water. The slurry in the centrifuge tube was shaken for one hour and then centrifuged at a speed of 1000 r/min for 10 min. To enrich the bacteria, 5 mL of the supernatant was added into a 250 mL conical flask containing 45 mL enrichment medium (0.5 g/L yeast extract, 5 g/L Na_2_HPO_4_·12H_2_O, 2.5 g/L KH_2_PO_4_, 5 g/L NH_4_Cl, 0.5 g/L MgSO_4_·7H_2_O) and incubated in a shaker (Jiangsu Jinyi Instrument Technology Co., Ltd, Changzhou, China) (100 r/min, 25 °C) for 48 h. Thereafter, 5 mL of the enriched bacteria was added into a 100 mL serum bottle containing 45 mL inorganic salt medium (5 g/L Na_2_HPO_4_·12H_2_O, 2.5 g/L KH_2_PO_4_, 5 g/L NH_4_Cl, 0.5 g/L MgSO_4_·7H_2_O) and BaP or BDE-47 (50 µg/L) and crimp-sealed with a Teflon-lined rubber septum, incubating in the shaker (100 r/min, 25 °C) for 21 d. To increase the biomass, 3 mL of the BaP or BDE-47 incubated bacteria were sampled and enriched with 27 mL enrichment medium in a 50 mL centrifuge tube for 96 h (100 r/min, 25 °C). Thereafter, the enriched bacteria were centrifuged and washed with the inorganic salt medium three times. The obtained bacteria were used for the degrading experiments.

Two sets of degrading (BaP degrading and BDE-47 degrading) experiments were set up using 20 mL vials. Each set contained 20 vials. Each vial contained 3.6 mL inorganic salt medium, 0.4 mL enriched bacteria and 2 mg/L BaP or BDE-47. All the vials were crimp-sealed with aluminum caps and incubated in the shaker (100 r/min, 25 °C). At day 0, 14, 21, and 28, two vials from each set were sacrificed for analyses of BaP or BDE-47 and their degradation products. At day 28, five vials from each set were sacrificed for analyses of bacterial community structures.

### 2.3. Analyses of BaP, BDE-47 and Their Degradation Products

For the extraction of BaP and its degradation products, a 4 mL culture from each vial was put into an extraction tube, 50 μL of 2 mg/L 10-deuterated pyrene (Pyr-d10) was added to the extraction tube as a recovery indicator, 4 mL n-hexane was added as the extraction agent, and the solution was vortexed for 4 min. After settling for 3 min, the upper organic phase was removed to a glass tube, and the water phase was extracted with 4 mL n-hexane. This procedure repeated three times. The organic phase was combined in the tube and anhydrous sodium sulfate was added to remove the residual water. The dehydrated organic phase was transferred to a nitrogen-blowing tube and blown dry with nitrogen. Then, 1 mL of n-hexane was added to redissolve the dried substances, solution was transferred to a 1 mL vial, and 50 μL of a 2 mg/L BaP-d12 was added as an internal standard. For the extraction of BDE-47 and its degradation products, a similar procedure as mentioned above was performed, except that PCB-141 was added as a recovery indicator and PCB-208 was added as an internal standard.

Agilent’s 6890 series gas chromatography (Santa Clara, CA, USA) and 5973N mass spectrometry system (Santa Clara, CA, USA) (GC-MS, equipped with a 0.25 mm × 30 m, film thickness 0.25 μm DB-5MS nonpolar gas chromatography column, EI source mass spectrometry detector) was used to measure BaP, BDE-47, and their degradation products. The gas chromatography working conditions were as follows: injection port temperature 280 °C, using splitless injection; carrier gas flow rate 1.5 mL/min; injection volume 1 μL; the initial column oven temperature was set at 110 °C, then raised to 180 °C at a rate of 8 °C/min, held at 180 °C for 1 min, then raised to 188 °C at a rate of 4 °C/min, held at 188 °C for 1 min, then raised to 210 °C at 4 °C/min, and finally raised to 305 °C at a rate of 20 °C/min and held at 305 °C for 5 min. The mass spectrometry working conditions was as follows: ion source temperature 246 °C; transfer line temperature 280 °C; ionization energy 70 eV; data collection method: selected ion monitoring (SIM). The mass-to-charge ratio of the quantitative and qualitative ions of the target substances was determined based on the mass spectrometry obtained from the full-scan standard. Identification was based on the retention time, the mass-to-charge ratio, and the abundance ratio of the fragment ions of the target substances.

Standard curves for BaP and BDE-47 were prepared at concentrations of 1, 2, 5, 10, 20, 50, 100, 200, 500, and 1000 μg/L. Standard curves for the possible reductive debromination products of BDE-47 (BDE28, BDE-17, BDE-15, BDE-7, BDE-4, BDE-3, BDE-1) were prepared at concentrations of 1, 2, 5, 10, 20, 50, 100, 200, and 500 μg/L. The detection limits for these compounds were all 1 μg/L. The extraction recoveries for these compounds ranged from 80% to 105%.

### 2.4. Analyses of Bacterial Community Structure

Both the soil used for inoculation and the two cultures were analyzed. About 0.5 g soil was used. For the cultures, a filtration device with a 0.22 μm cellulose acetate membrane was used to filter the bacteria. PowerSoil^®^DNA kit (MIBIO, Nashville, TN, USA) was employed to extract DNA from the soil or the bacteria-coated membrane. The integrity of DNA was detected by 1% agarose gel electrophoresis. The concentration and purity of DNA were determined by ultramicro UV spectrophotometer (NanoDrop one, Madison, WI, USA). Bacterial specific primers 338F (5′-ACTCCTACGGGAGGCAGCA-3′) and 806R (5′-GGACTACHVGGGTWT-CTAAT-3′) with 12 bp barcode were used to amplify 16S rRNA gene in V3-V4 region [20,21]. PCR products were mixed in equidense ratios according to the GeneTools Analysis Software (Version4.03.05.0, SynGene, Bengaluru, Karnataka, India), and the mixed PCR products were purified with an E.Z.N.A Gel Extraction Kit (Omega Bio-tek, Radnor, PA, USA). The NEBNext^®^ Ultra™ DNA Library Prep Kit for Illumina^®^ standard procedures (New England Biolabs, Ipswich, MA, USA) were followed for library construction. The constructed amplicon library was sequenced for PE250 using Illumina Hiseq2500 platform. After data filtering, clean reads stitching, and quality filtering, the effective Clean Tags were obtained. All Clean Tags of all samples were clustered by USEARCH software (V8.0.1517, drive5, San Francisco, CA, USA). The sequences were clustered into OUT (Operational Taxonomic Units) with 97% identity by default, and the default clustering method was UPARSE. The sequence with the highest frequency was used as the representative sequence of each OTU for subsequent annotation to obtain the community composition information of each sample. Qiime (V1.9.1, Knight Lab at the University of Colorado, Boulder, CO, USA) and R (V2.15.3, Statistics Department of the University of Auckland, Auckland, New Zealand) were used to analyze and map the bacterial community data.

## 3. Results and Discussion

### 3.1. Degradation of BaP by the Cultured Microorganisms

**Figure 1:**
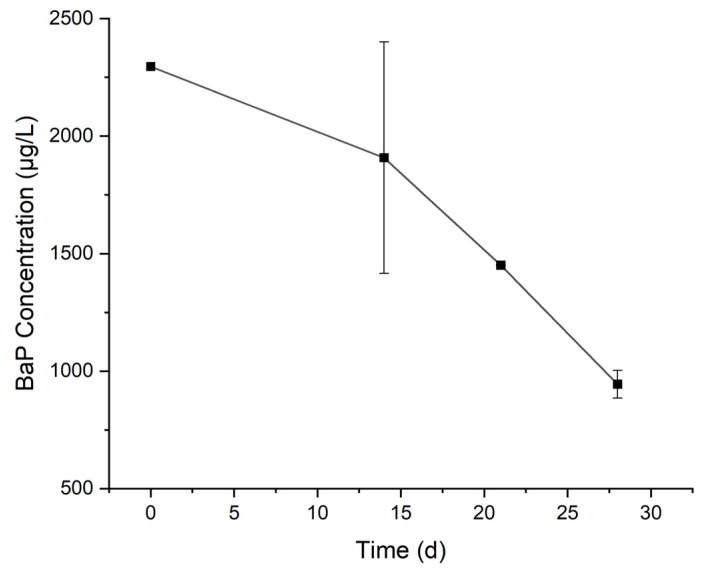
Change of BaP concentration over time in the cultured medium.

The change in BaP concentration over time in the culture medium is shown in Figure 1. In 28 days, the concentration of BaP decreased from 2.30 mg/L to 0.94 mg/L. By fitting with the first-order reaction model (R^2^ = 0.878), the obtained rate constant of degradation was 0.030 /d and the half-life was 23.1 d. Serum bottles sealed with aluminum crimps were used to culture the microorganisms to avoid the volatilization of BaP. Therefore, the observed decrease in BaP in the culture medium was mainly due to biodegradation. Growth of microorganisms over time was observed visually as the culture medium gradually became turbid. In the culture medium, BaP was provided as the sole carbon source, indicating that the cultured microorganisms could grow on BaP. The degradation rate constant was within the range (0.006–0.051/d) reported in the literature [22]. The incubation for degradation was presumably aerobic. In a nominal 20 mL vial, the actual total volume of a crimped closed (rubber septum) vial is closer to 25 mL. With 4 mL of medium there was about 20 mL of gas phase, and the amount of O_2_ was 0.13 mmol (4.16 mg). This ought to be in good excess over BaP which was about 8 µg/vial (0.0317 µmol).

**Figure 2:**
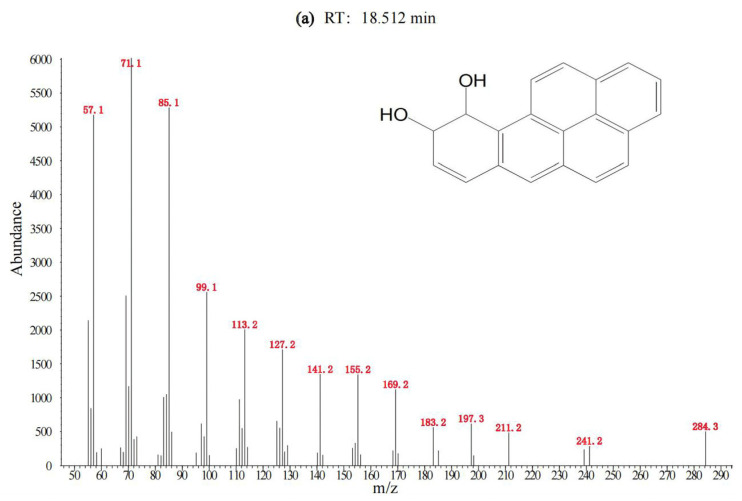

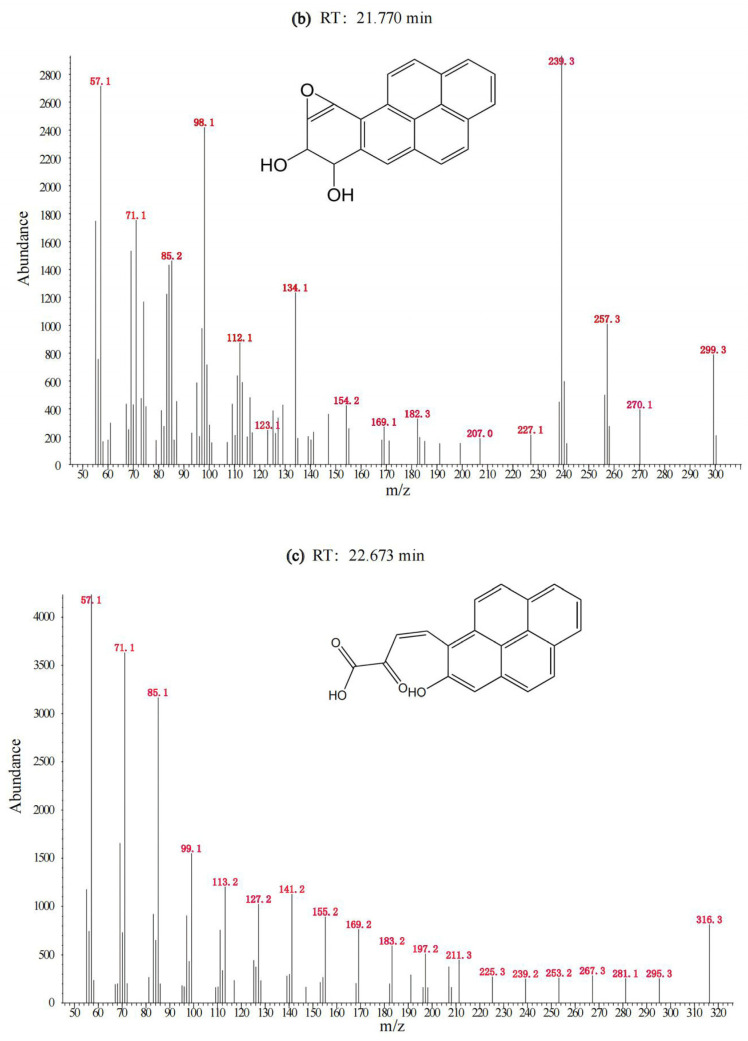
Degradation products of BaP identified by GC-MS using full scan mode. (**a**) benzo[a]pyrene-4,5-diol or its isomers; (**b**) benzo(a)pyrene-7,8-dihydrodiol-9,10-epoxide; (**c**) cis-4 (8-hydroxypyrenyl-7) -2-oxo-3-butenoic acid.

**Figure 3:**
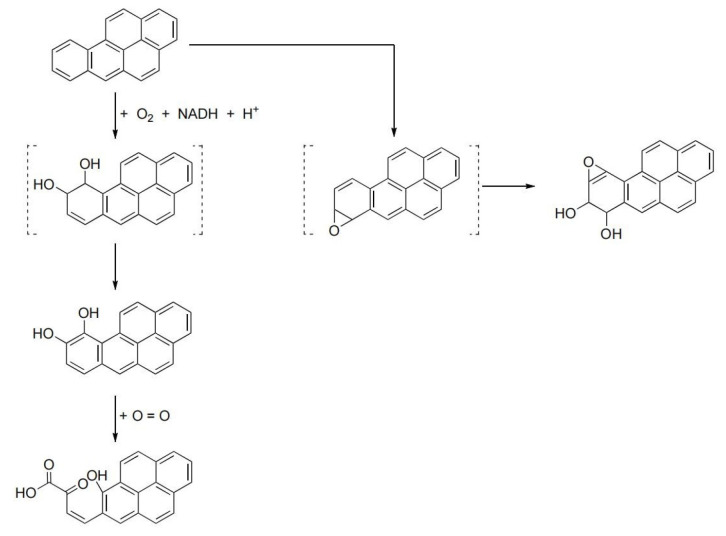
Proposed degradation pathway of BaP by the cultured soil microorganism (compounds in dashed parentheses are speculated compounds; arrow lines indicate the direction of degradation).

To identify the degradation products of BaP, samples collected at different days were analyzed by GC-MS using full scan mode. At day 7, day 14, and day 21, three degradation products were identified, i.e., benzo[a]pyrene-9,10-dihydrodiol or its isomers (benzo[a]pyrene-4,5- dihydrodiol, benzo[a]pyrene-7,8- dihydrodiol, benzo[a]pyrene-11,12- dihydrodiol), benzo(a)pyrene-7,8-dihydrodiol-9,10-epoxide, and cis-4 (8-hydroxypyrenyl-7)-2-oxo-3-butenoic acid, respectively, as shown in Figure 2. Due to lack of standards and limited samples, the changes in these products over time were not monitored. The proposed degradation pathway of BaP is shown in Figure 3.

BaP can be biodegraded via a variety of pathways. In the study of Moody et al. [23], *Mycobacterium vanbaalenii* PYR-1 initially oxidized BaP with dioxygenases and monooxygenases at C-4,5, C-9,10, and C-11,12. The major intermediates of BaP metabolism that had accumulated in the culture media after 96 h of incubation were benzo[a]pyrene cis-4,5-dihydrodiol, (benzo[a]pyrene cis-11,12-dihydrodiol, benzo[a]pyrene trans-11,12-dihydrodiol, 10-oxabenzo-[def]chrysen-9-one, and hydroxymethoxy and dimethoxy derivatives of BaP. The degradation by *Citrobacter* sp. HJS-1 produced BaP-cis-7,8-dihydrodiol intermediate, and the initial oxidation mode and the binding site of BaP were revealed in the dioxygenase [24]. In the study of Dou et al. and Hu et al., putative biodegradation pathways were proposed with hydrogenation reduction as the initial BaP activating reaction under anaerobic condition [25,26]. In this study, oxidized products benzo[a]pyrene-9,10-dihydrodiol, benzo(a)pyrene-7,8-dihydrodiol-9,10-epoxide, and cis-4 (8-hydroxypyrenyl-7)-2-oxo-3-butenoic acid were detected. It was reported that as the benzene rings of BaP were cleaved, the toxicity of the metabolites gradually decreased [27]. On the other hand, benzo(a)pyrene-7,8-dihydrodiol-9,10-epoxide was shown exhibiting higher toxicity than BaP [28]. Therefore, it is important to monitor the degradation products of BaP, since the pathway and extent of BaP degradation determine the toxicity.

### 3.2. Degradation of BDE-47 by the Cultured Microorganisms

**Figure 4:**
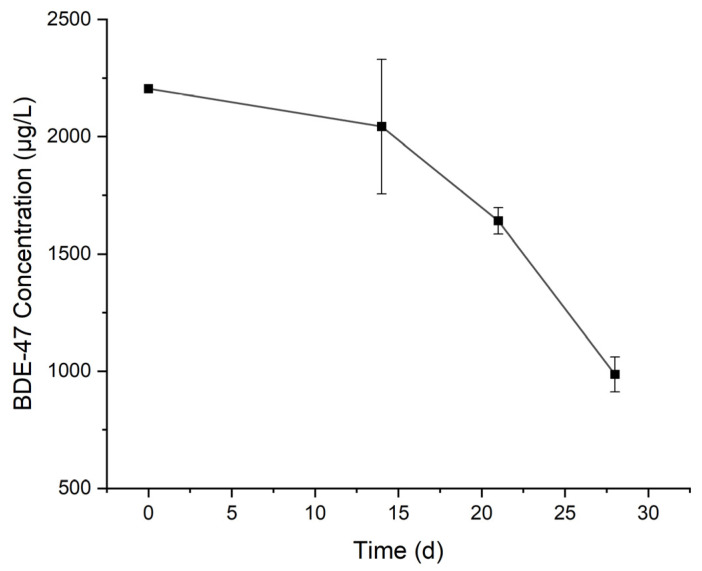
Change in BDE-47 concentration over time in the culture medium.

The change in BDE-47 concentration over time in the culture medium is shown in Figure 4. In 28 days, the concentration of BDE-47 decreased from 2.20 mg/L to 0.99 mg/L. By fitting with the first-order reaction model (R^2^ = 0.751), the obtained rate constant of degradation was 0.026 /d and the half-life was 26.7 d. Similar to BaP, serum bottles sealed with aluminum crimps were used to culture the microorganisms to avoid the volatilization of BDE-47. Therefore, the observed decrease in BDE-47 in the culture medium was mainly due to biodegradation. In the culture medium, BDE-47 was provided as the sole carbon source, and the growth of microorganisms over time was observed visually as the culture medium gradually became turbid. The obtained rate constant of BDE-47 degradation by the culture was lower than those (0.073 and 0.051 /d) by bacteria isolated from real e-waste sites [17]. The reason might be the initial concentration of BDE-47 (0.115 mg/L) in the above literature was much lower than that in this study (2.30 mg/L). Our previous study showed that the degradation rate of BDE-47 decreased with the increase in the initial concentration of BDE-47 [29]. The incubation for degradation was presumably to be aerobic. The amount of O_2_ 0.13 mmol (4.16 mg) was in good excess over BDE-47 which was about 8 µg/vial (0.0165 µmol).

**Figure 5:**
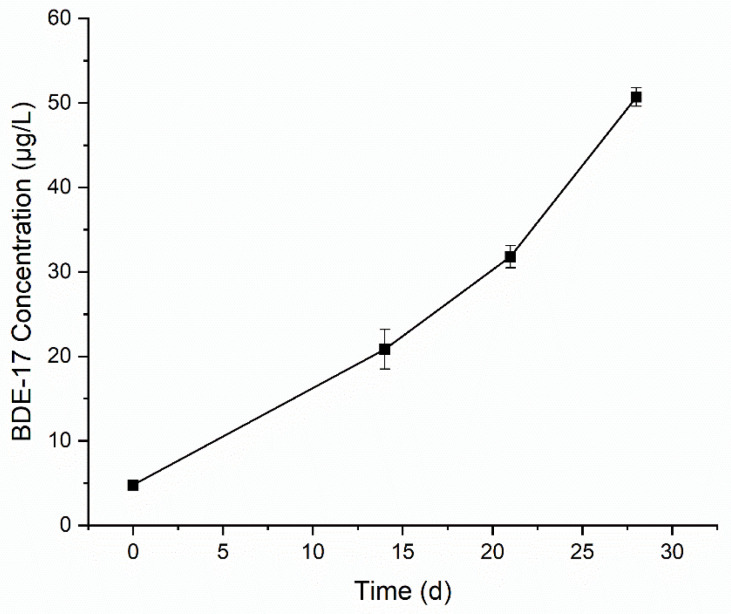
Change in BDE-17 concentration over time in the culture medium.

**Figure 6:**
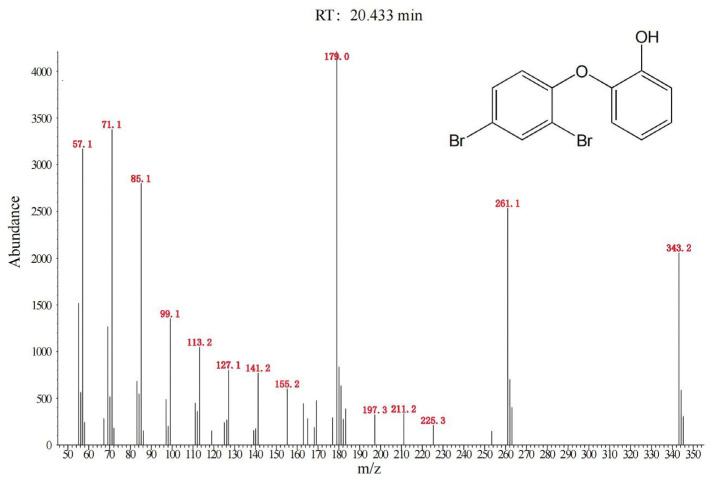
Degradation product of BDE-47 identified by GC-MS using full scan mode (hydroxylated dibromodiphenyl ether).

**Figure 7:**
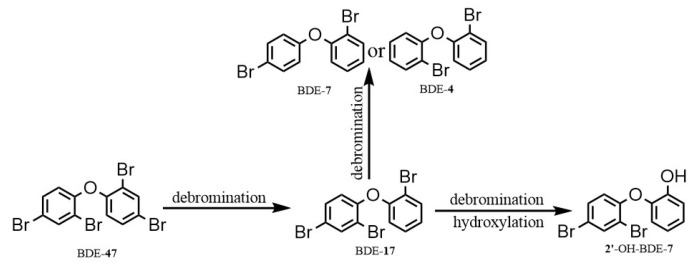
Proposed degradation pathway of BDE-47 by the soil culture microorganism.

The potential reductive debromination products of BDE-47 (BDE-28, BDE-17, BDE-15, BDE-7, BDE-4, BDE-3, and BDE-1) were measured. BDE-17 was detected, with concentration increasing from 0.005 mg/L at day 1 to 0.051 mg/L at day 28 (Figure 5). Trace amounts of BDE-7 were detected at day 21 (0.001 mg/L) and day 28 (0.003 mg/L). No other debromination products were detected. The productions of BDE-17 and BDE-7 accounted for about 5.4% of the reduction of BDE-47. To further identify the degradation products, samples were analyzed by GC-MS using full scan mode. At day 21, an oxidative degradation product, hydroxylated dibromodiphenyl ether, was detected. This is shown in Figure 6. Due to lack of standard limited samples, the change in this product over time was not monitored. The proposed degradation pathway of BDE-47 is shown in Figure 7.

BDE-47 can be biodegraded via both oxidative and reductive pathways [29,30]. In this study, both oxidative and reductive reactions occurred. Hydroxylated dibromodiphenyl ether such as 2′-OH-BDE-7 was the only oxidative product detected. The detection of hydroxylated daughter products of BDE-47 such as 6-OH-BDE-47, 5-OH-BDE-47, 2′-OH-BDE-28 and 4′-OH-BDE-17 has been reported in the literature [30,31]. This was the first time that hydroxylated dibromodiphenyl ether has been identified.

### 3.3. Characterization of BaP and BDE-47 Degrading Bacteria in the Culture Medium

**Figure 8:**
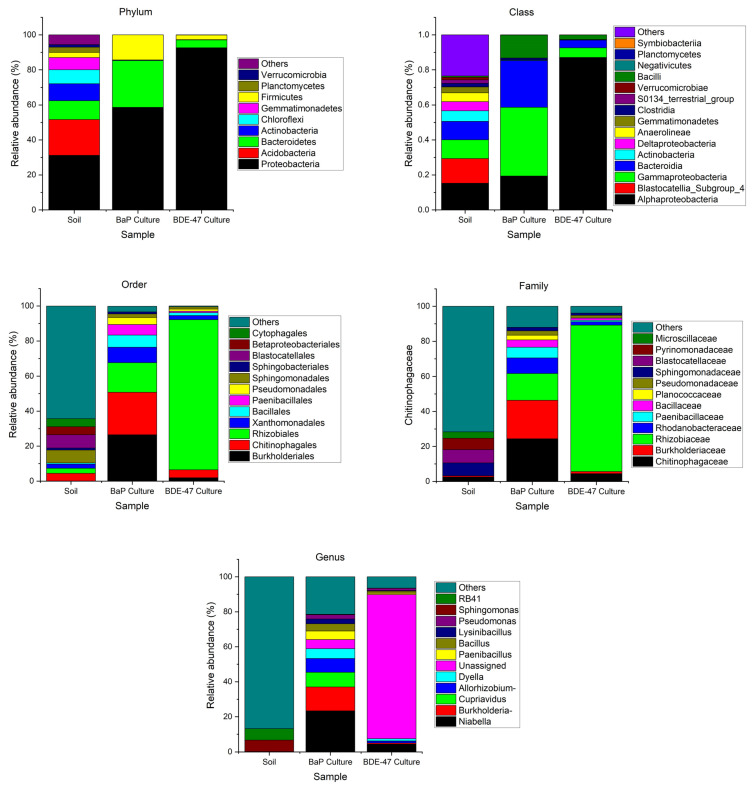
Comparison of bacterial community structures among different samples (soil: original soil used to inoculate the cultures; BaP culture: culture enriched with BaP as the sole carbon source; BDE-47 culture: culture enriched with BDE-47 as the sole carbon source; Allorhizobium: refers to Allorhizobium-Neorhizobium-Pararhizobium-Rhizobium; Burkholderia: refers to Burkholderia-Caballeronia-Paraburkholderia; the levels of bacterial community structures are shown on the tops of subfigures).

The comparison of the bacterial community structures among samples collected from the soil used as an inoculum and the two enriched cultures (at day 28) are shown in Figure 8. The richness and diversity of bacteria in the two cultures were much less than that in the soil. The BaP culture had higher richness and diversity than the BDE-47 culture. The bacterial community structures were quite different among the three samples. In the BDE-47 culture, an unassigned species in the Rhizobiaceae (Rhizobiales, Alphaproteobacteria, Proteobacteria) accounted for 82.3% of the total species. This unassigned species was detected in the BaP culture at lower abundance (5.2%) and trace in the soil (<0.01%). In the BaP culture, the top nine genera were Niabella (23.4%, belonging to Chitinophagaceae, Chitinophagales, Bacteroidia, Bacteroidota), Burkholderia-Caballeronia-Paraburkholderia (13.7%) and Cupriavidus (8.3%) (both belonging to Burkholderiaceae, Burkholderiales, Gammaproteobacteria, Proteobacteria), Allorhizobium-Neorhizobium-Pararhizobium-Rhizobium (8.0%, belonging to Rhizobiaceae, Rhizobiales, Alphaproteobacteria, Proteobacteria), Dyella (5.6%, belonging to Rhodanobacteraceae, Xanthomonadales, Gammaproteobacteria, Proteobacteria), Paenibacillus (4.9%, belonging to Paenibacillaceae, Paenibacillales, Bacilli, Firmicutes), Bacillus (4.2%, belonging to Bacillaceae, Bacillales, Bacilli, Firmicutes), Lysinibacillus (2.7%, belonging to Planococcaceae, Bacillales, Bacilli, Firmicutes), and Pseudomonas (2.5%, belonging to Pseudomonadaceae, Pseudomonadales, Gammaproteobacteria, Proteobacteria). These species were slightly enriched in the BDE-47 culture (0.04–4.4%) and hardly detected in the original soil (<0.01%). In the original soil, Sphingomonas (Sphingomonadaceae, Sphingomonadales, Alphaproteobacteria, Proteobacteria) was a major species (6.5%). It was also detected in the BaP culture and BDE-47 culture, but at lower abundances (0.2% and 0.1%). Other major species such as RB41 were not detected in the two cultures.

Previous studies showed that some species of Bacteria were able to degrade BaP and BDE-47. Aitken et al. isolated 11 strains from a variety of contaminated sites (oil, motor oil, wood treatment, and refinery) with the ability to degrade BaP. The organisms were identified as at least three species of *Pseudomonas*, as well as *Agrobacterium*, *Bacillus*, *Burkholderia* and *Sphingomonas* species [32]. BaP has also been reported to be degraded by other bacteria including *Rhodococcus* sp., *Mycobacterium*, and mixed culture of *Pseudomonas* and *Flavobacterium* species [33,34,35]. Isolated from an e-waste-contaminated site, strain B. *cereus* S1 could use BDE-47 as the sole carbon source under aerobic condition, and strain A. *faecalis* S4 could use BDE-47 as the sole carbon source under anaerobic condition [17]. BDE-47 could also be degraded by *Acinetobacter pittii* GB-2 [30]. In this study, *Pseudomonas*, *Bacillus, Burkholderia* and *Sphingomonas* were detected in the BaP and BDE-47 cultures at varied abundances. The other reported BaP and BDE-47 degrading bacteria were not detected in this study. However, some other bacteria were enriched in the presence of BaP (such as *Niabella*, *Cupriavidu*, *Allorhizobium-Neorhizobium-Pararhizobium-Rhizobium*, *Dyella*, *Paenibacillus*, and *Lysinibacillus*) and in the presence of BDE-47 (such as an unassigned species in the Rhizobiaceae). Further studies are needed to confirm the abilities of these bacteria to degrade BaP or BDE-47.

## 4. Conclusions

In the culture with BaP or BDE-47 as the sole carbon source, the degradation of BaP or BDE-47 occurred at first-order rate constants of 0.030/d or 0.026/d. For BaP, the degradation products included benzo[a]pyrene-9,10-dihydrodiol or its isomers, benzo(a)pyrene-7,8-dihydrodiol-9,10-epoxide, and cis-4 (8-hydroxypyrenyl-7)-2-oxo-3-butenoic acid. For BDE-47, the degradation products included BDE-17, BDE-7, and hydroxylated dibromodiphenyl ether. The bacterial community structures among the soil used as an inoculum, the BaP culture, and the BDE-47 culture were quite different. The richness and diversity of bacteria in the two cultures were much less than those in the soil. The BaP culture had higher richness and diversity than the BDE-47 culture. In the BDE-47 culture, an unassigned species in the Rhizobiaceae was dominant (82.3%). In the BaP culture, multiple species such as *Niabella* (23.4%), *Burkholderia-Caballeronia-Paraburkholderia* (13.7%), *Cupriavidus* (8.3%), *Allorhizobium-Neorhizobium-Pararhizobium-Rhizobium* (8.0%), etc. were dominant.

## Data Availability

The data presented in this study are available on request from the corresponding author. The data are not publicly available due to privacy.
